# Riedel's thyroiditis as a diagnostic dilemma - A case report and review of the literature

**DOI:** 10.1016/j.amsu.2020.02.006

**Published:** 2020-02-25

**Authors:** Alam Ara Shafi, Nourah Bin Saad, Bandar AlHarthi

**Affiliations:** King Fahad Medical City, Makkah Road, Riyadh, 11525, Saudi Arabia

**Keywords:** Riedel's thyroiditis, Thyroid mass, Anaplastic carcinoma, Thyroid lymphoma, Diagnostic challenges

## Abstract

Riedel's thyroiditis is a rare inflammatory process which not only involves thyroid gland but also the surrounding vital structures. It may also be associated with various forms of systemic fibrotic disorders. The exact etiology is not known, but currently, the most favored view is that of a localized form of the systemic fibrotic process. We report a case of Riedel's thyroiditis in a male patient, highlighting diagnostic challenges and a rare presentation of hypocalcemia and mimicking thyroid lymphoma. Clinical knowledge of such a presentation of Riedel's thyroiditis would enhance our ability to make a speedy diagnosis. Apart from avoiding aggressive surgical intervention, awareness of such a clinical entity may avoid complications and hence morbidity. Our case also highlights the difficulty in histological diagnosis which is vital to rule out malignancy and avoiding any major surgical intervention fraught with complications. Although the patient had a poor tolerance to Tamoxifen and Rituximab, however, his response to high dose steroids is the currently accepted treatment of choice. This case adds to the sparse literature available on the cytological diagnosis of RT and highlights the diagnostic challenge due to suspicious radiology findings.

## Highlights

• Riedel's thyroiditis, a rare form of chronic thyroiditis is associated with a fibrotic process involving the thyroid gland, leading to anatomical and physiological consequences.
• Riedel's thyroiditis is part of the spectrum of IgG4-related systemic disorders (IgG4-RSD).
• Clinicians should be aware of RT to differentiate this condition from other thyroid disorders, especially malignant lesions.
• Thyroidectomy is indicated for patients with compressive symptoms, suspicion of malignancy and failure of medical management.

## 1 Introduction

Riedel thyroiditis (RT) is a rare fibro sclerotic condition affecting the thyroid gland, characterized by thyroid parenchymal replacement with fibrous tissue. It was first described by Bernhard Riedel in 1896. He described 3 cases of a peculiarly hard, infiltrative lesion of the thyroid gland [1,2]. until now, there have been almost 200 cases reported in the literature. This sclerotic process may not be limited to the thyroid only, but invades the surrounding vital structures like vessels, nerves, trachea, esophagus, and parathyroid that leads to compressive symptoms and endocrine abnormalities. RT is found in only 0.06% of all thyroidectomies, and reports are often limited to case reports and small case series [3]. At the Mayo Clinic, over a period of 64 years until 1985, only 37 patients with RT were encountered (out of 56,700 patients who underwent thyroidectomy) [4], highlighting the rarity of the disease, with an estimated incidence of 1.06 cases per 100,000 outpatients, accounting for 0.06% and 0.05% of thyroidectomies in two separate series at the Mayo Clinic, London tertiary healthcare level [4,5]. Just like other thyroid disorders the RT is more common in women, with a recent case series showing 81% of confirmed Riedel's diagnoses from 1976 to 2008 were in women [6].

The exact etiology of the condition is not known but the current view favors it to be a local manifestation of a systemic fibrotic process or an autoimmune process [7]. It has long been linked to a generalized fibroinflammatory process termed “multifocal fibrosclerosis” (MFS) [8]. RT appears to be the thyroid disease with the strongest association to IgG4-RSD.

Our search for medical literature on PubMed and EMBASE database revealed that no report of Riedel's thyroiditis has been made in the Middle East and specifically in Saudi Arabia yet. We report this case of a 35-year-old Saudi man presented with clinical features initially suggestive of malignant goiter with the possibility of primary thyroid lymphoma, which turned out to be Riedel's thyroiditis after histological examination of the excised thyroid specimen.

The report highlights the difficulties that one may encounter in terms of diagnosing and managing such a case. As per our knowledge, this is the first report of a case of Riedel's thyroiditis in the Middle East.

## 2 Case report

**Fig. 1:**
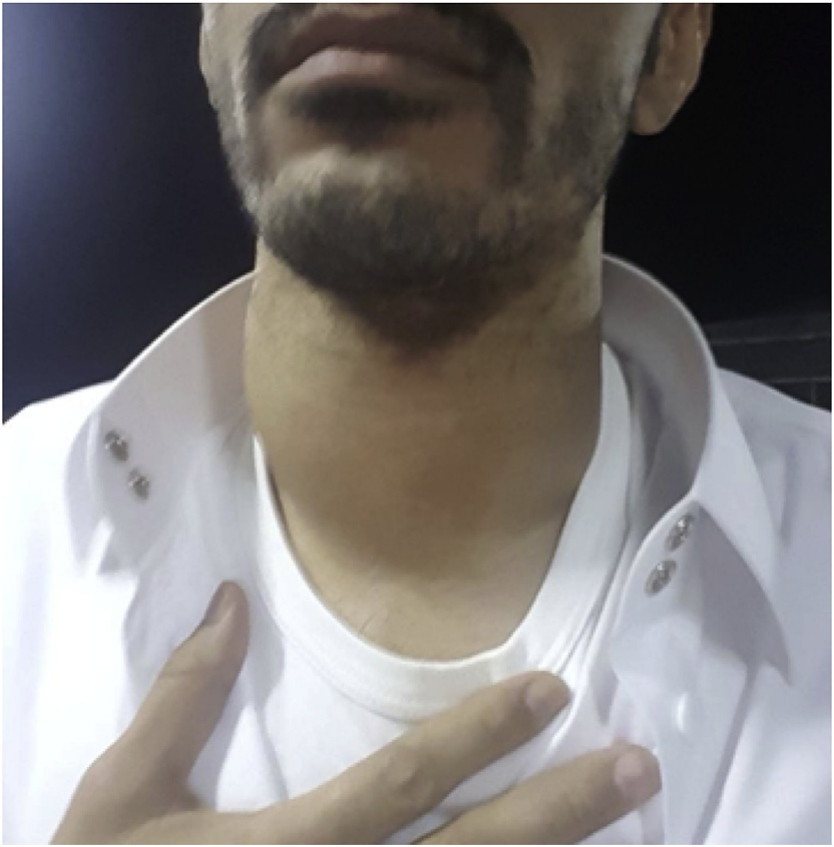
Diffuse enlargement of thyroid at Presentation [more on right side]: Anterior view.

A 35 years old man of Najran district of Saudi Arabia was referred to the Endocrine Surgery Division of Surgical Oncology Department at King Fahad Medical City, Riyadh, as a case of diffuse goiter, symptomatic hypothyroidism, and hypocalcemia. His main complaint was a one-year history of painless progressive anterior neck swelling which rapidly increased in size during the four months before presentation. It was associated with the feeling of chocking and dysphagia for large boluses of food and occasional dyspnea. There was a significant weight loss of 20 Kg with a recurrent low-grade fever for the last six months. The systemic review was positive for eyelid swelling and excessive lacrimation. The past history was not significant for any chronic illness or surgery. He uses to smoke one packet of cigarettes per day for the last 15 years. He was on Thyroxin 125 mcg daily, Alfacalcidol 1mcg daily and calcium supplements as per his primary care physician prescription. Physical examination revealed a middle-aged well-nourished man with normal vital signs as per his age. There was a diffusely enlarged thyroid gland measured about 15cm and 10cm in transverse and vertical axis respectively [Fig. 1] with a smooth surface and normal overlying skin. There were multiple, small, firm and mobile enlarged cervical lymph nodes. The carotid pulsation was palpable bilaterally.

Thyroid function test showed thyroid stimulating hormone (TSH) level of 0.903 IU/mL (reference: 0.4–4.0), a normal free thyroxin (fT4) level of 17 pmol/L (reference: 9–22.2). Full blood count result showed white blood cell count of 7800 cells/cmm (reference: 4000–11,000) with neutrophils 43% (reference: 40–70), lymphocytes 54% (40–60) and eosinophils of 3% (reference: <2), platelets count was 109 × 103/cmm (reference: 150,000–450,000) and packed cell volume of 41% (reference: 38–48). Antuthyroid antibodies; Tg: 420 IU/ml (reference: 0–4.11) TPO: 567 IU/ml (reference: 0–5.61). IgG4 levels were normal.

**Fig. 2:**
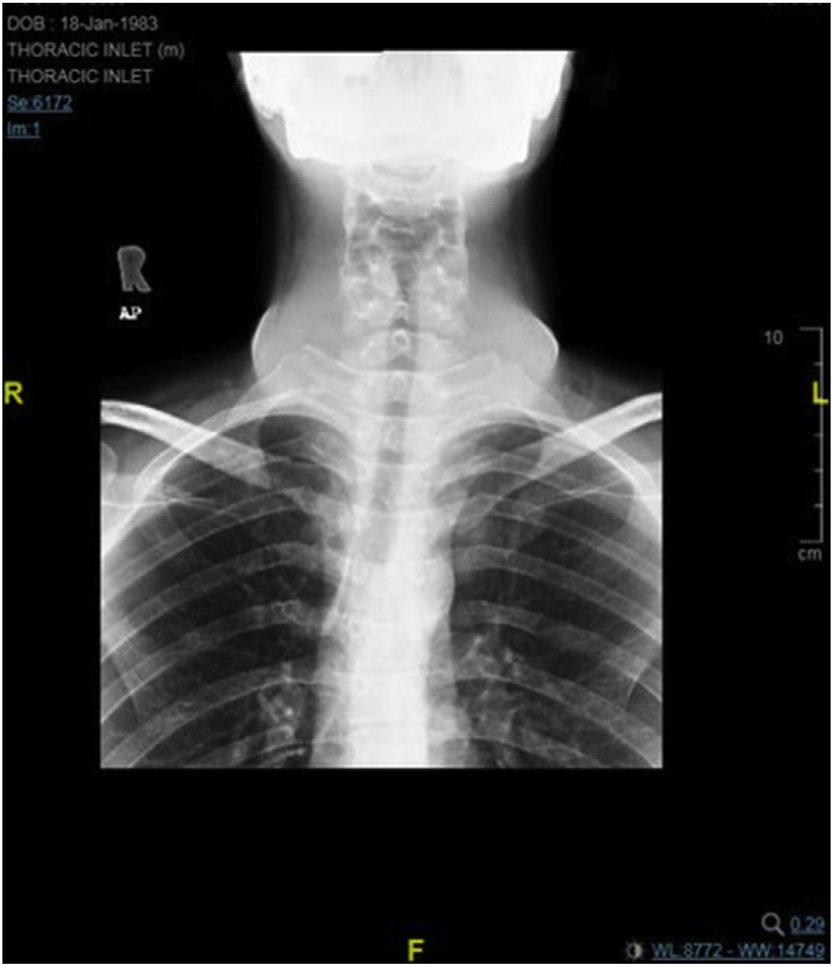
Chest X-Ray (Antero-posterior view) showed diffuse swelling in the neck with patent none deviated trachea.

**Fig. 3:**
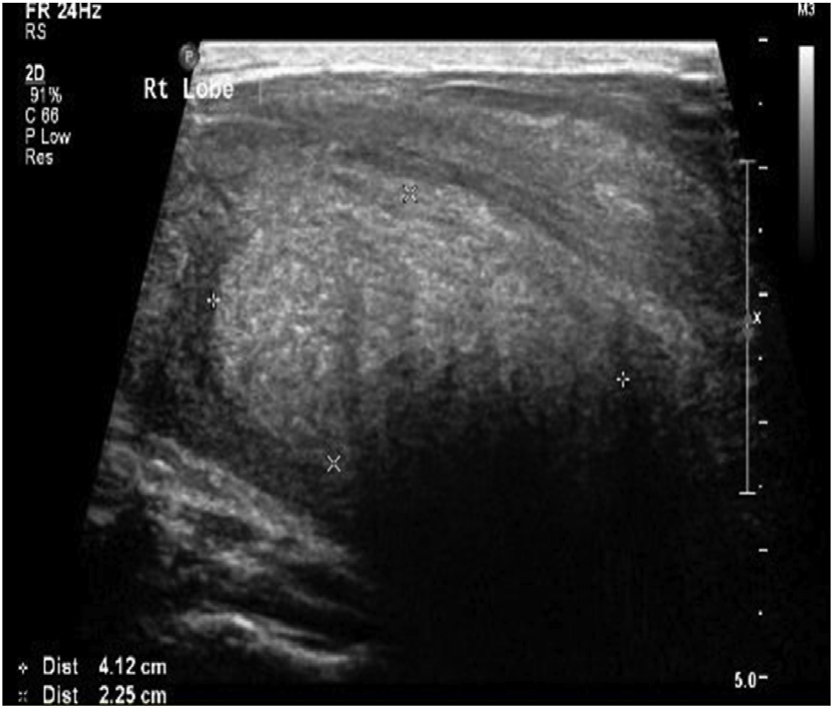
Ultrasound scan of the neck revealed Heterogeneous parenchymal echotexture is seen of both the thyroid lobes with multiple heterogeneous nodules seen. Couples of subcentimeter lymph nodes are seen in the bilateral cervical recesses.

**Fig. 4:**
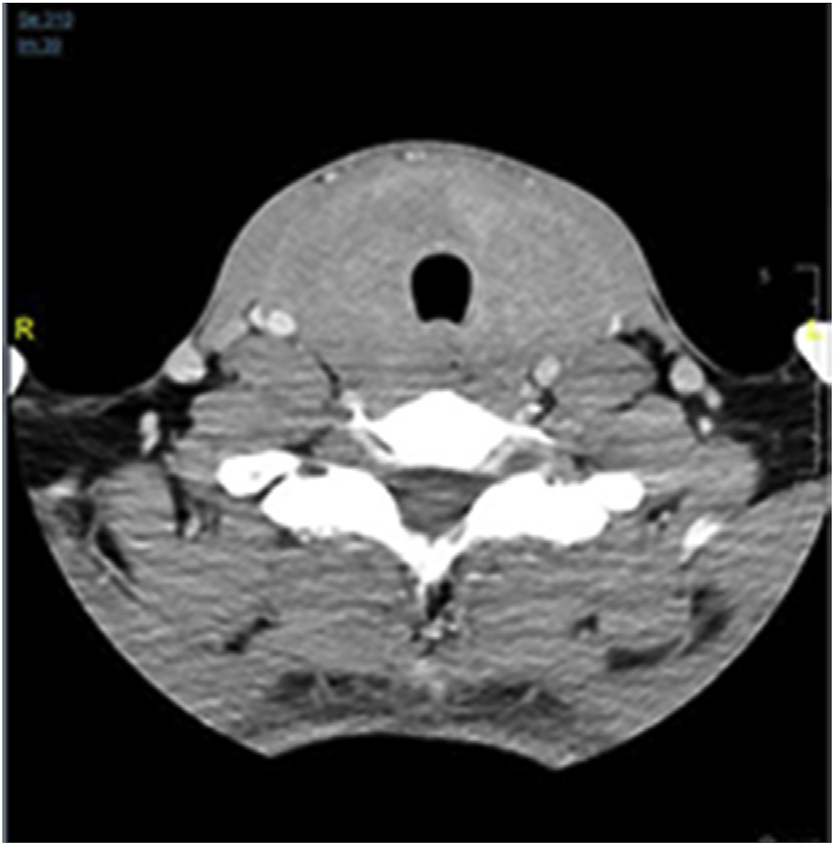
CT-Scan neck showed diffusely enlarged thyroid gland with heterogeneous enhancement, no calcification and minimal retrosternal extension. There was no tracheal compression or displacement but both common carotids and internal jugular veins were displaced posterolaterally. Multiple small suspicious lymph glands were noted at level II, III, IV and paratracheal region bilaterally.

Radiology [Fig. 2, Fig. 3, Fig. 4] demonstrated the nonspecific thyroid enlargement with cervical lymphadenopathy. Initial Fine-needle aspiration cytology (FNAC) from both thyroid lobes was inadequate. Repeat FNAC thyroid showed scanty lymphocytes likewise the FNAC from cervical lymph nodes. A clinical diagnosis of primary thyroid lymphoma was made. Further immunohistochemistry staining and flow cytometry did not confirm our clinical diagnosis of thyroid lymphoma.

Keeping in view his symptomatology, he was scheduled for a total thyroidectomy. Upon neck exploration via a classic collar incision, it was difficult to find the resectional planes due to diffuse fibrotic infiltration of the strap muscles and surrounding soft tissues. The whole of the thyroid gland was hard and infiltrative to surrounding tissues.

**Fig. 5:**
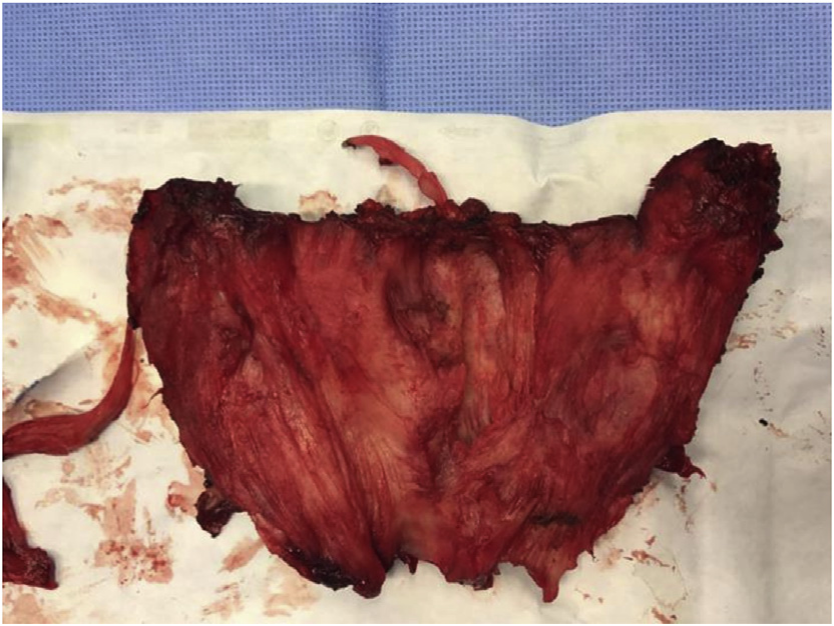
Surgical specimen.

An intraoperative frozen section examination was performed due to suspicion of thyroid malignancy. It was reported as fibrous tissue with spindle cells, in favor of Riedel's thyroiditis. We performed isthmusectomy, Partial right lobectomy and excision of a small quantity of the left lobe that could be safely resected for histopathological examination [Fig. 5]. The bulk of the tumor mass could not be resected due to diffuse extrathyroidal infiltration into the surrounding vital structures, so further surgical resection was aborted to prevent fatal complications. It was technically a challenging surgery so a Jackson-Pratt drain number 12 was left in the wound in a sub-fascial plan.

**Fig. 6:**
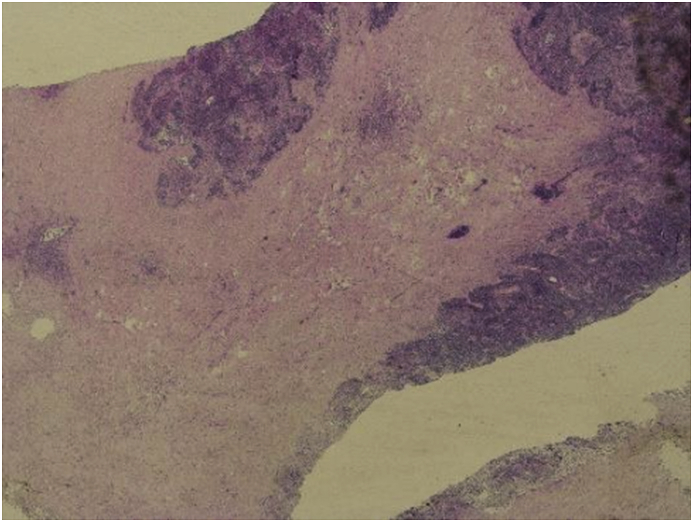
Fibrosis and inflammation.

**Fig. 7:**
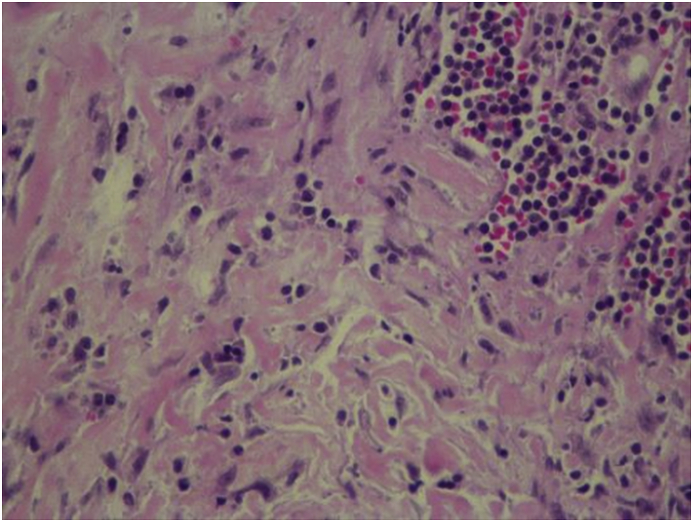
High power view of the surgical pathology specimen showing extensive bands of fibrosis with plasma cells and lymphocytic infiltration consistent with Riede's Thyroiditis (Hematoxylin-Eosin stain) Lymphoplasmacytic infiltrate.

**Fig. 8:**
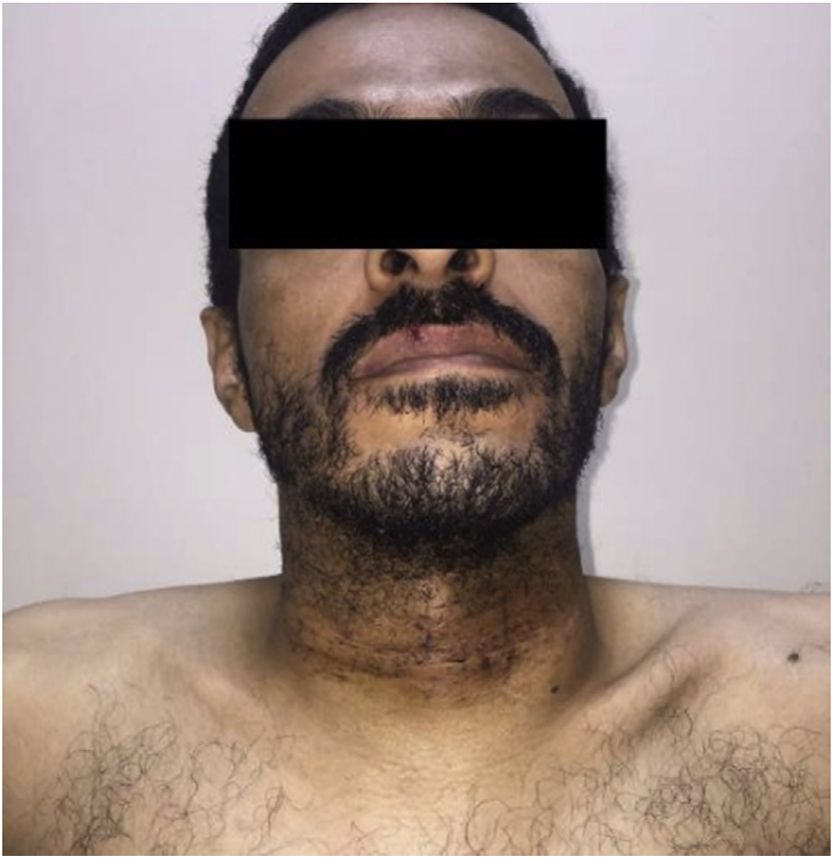
Patient two weeks post-surgery.

He had an uneventful postoperative course without any voice changes or swallowing difficulties. Histopathology of the thyroid gland showed diffuse and extensive collagenous fibrosis with marked infiltration of chronic inflammatory cells and blood vessels were damaged by the extensive fibrosis [Fig. 6, Fig. 7]. No thyroid or parathyroid tissue was identified in the resected specimen. Immunohistochemical staining with various markers was performed for differential diagnosis with the following results: Vimentin (þ), LCA (þ), CD68 (þ), CK5/6 (squamous metaplasia nestþ), Ki-67 (þ), Syn (--), CT (--), TG (--). Taken together, Riedel's thyroiditis was the final pathological diagnosis. After the endocrine team review, he was started on Tamoxifen 20 mg daily, and Prednisolone 5 mg daily with the continuation of Thyroxin 125 mcg/day and calcium carbonate and Alfacalcidol 0.5 μg daily. The patient was discharged from the hospital on 6th postoperative day after removal of the drain [Fig. 8].

After four months of Tamoxifen use, he develops bilateral internal jugular vein and intracerebral venous thrombosis (sigmoid sinus) that was treated successfully with 3 months use of enoxaparin, Tamoxifen was stopped and Rivaroxaban 20 mg daily was added to his treatment regime with the continuation of Prednisolone. He was started on Rituximab but he did not tolerate it and the drug was discontinued. His pan CT-scan showed no abnormality in his chest and abdomen and his current CT-scan neck showed the stability of the thyroid swelling for the last two years. He is following with rheumatology, endocrinology, and ophthalmology. His current medications are, Thyroxin 125 μg/day, Vitamin D 50,000 IU/month, calcium carbonate 1 gm/day, Prednisolone 5 mg/day and Rivaroxaban 20 mg/day.

## 3 Discussion

RT is well known for its rarity and until now few cases have been reported in the medical literature. This reported case of Riedel thyroiditis is the first in the history of over 15 years' existence of our hospital with over 100 thyroidectomies being performed per year. This reflects a hospital incidence rate of about 1 in 1500 (0.067) cases of thyroidectomies. This figure is found to be similar to reports from other centers of the world [4,5].

Peak age incidence for RT is in the fifth decade and women are mostly affected with a female to male ratio of 4:1 [6]. Reported patient being a male in his mid-forth decade does not coincide with the peak age and gender distribution. This also does not coincide with the age distribution of thyroid malignancy including primary thyroid lymphoma [9,10]. However a recent study by Falhammar et al. have reported a similar case of 32 years old male patients among their case series of six patients [11].

There have been many postulations regarding the etiopathogenesis of RT. These include “intrathyroidal hypothesis" claiming late-stage chronic inflammatory disorder of thyroid; "pharmacological hypothesis" suggesting the implication of certain medications as a trigger of RT and “systemic autoimmune hypothesis” (multifocal fibrosclerosis hypothesis) based on the association of RT with other fibro-inflammatory conditions like retroperitoneal fibrosis, mediastinal fibrosis, sclerosing cholangitis [12,13]. Currently systemic autoimmune hypothesis has the most support and RT is now considered to be an IgG4‐RSD. Although our patient did not have the manifestations of any other fibrotic disorder and IgG/IgG4 staining was not performed in the tissue section, however; the serum levels of IgG/IgG4 were elevated (435 mg/dl). More recently attention has been focused on the role of gene expression in the etiology and pathogenesis of RT. Wojciechowska-Durczynska et al. studied the quantitative analysis of thyroid tissue of RT patients for gene expression and found that *PIK3CA* (responsible for coding alpha catalytic subunit of class 1 *PI3K* phosphoinositide 3-kinase and *CDH3* responsible for coding P-cadherin) gene expression level was higher than respective control of normal thyroid tissue [24]. Phosphoinositide 3-kinase/serine-threonine protein kinase *(PI3K/Akt)* pathway which participates in cellular signaling in response to various growth factors including fibroblast growth factor, when genetically activated and amplified lead to enhancement and stimulation *PIK3CA* kinase activity and Akt phosphorylation [14].

As there are no features pathognomonic to RT, the majority of case reports in the past have a similar clinical presentation like the current case [4,6,11]. Consequently, primary thyroid lymphoma, Hashimoto's thyroiditis, and anaplastic carcinoma were among our differential diagnosis; keeping in view our patient's initial presentation with a hard fixed thyroid mass, FNA results, enlarged cervical nodes, and presence of hypothyroidism. However, the extent of fibrosis in these disorders is much less and doesn't extend beyond the thyroid [1]. Cervical lymphadenopathy is usually not present in RT but has been reported [15]. The finding of inflammatory cell infiltrate mainly lymphocytes in this patient is suggestive of lymphadenopathy was may be due to reactive inflammation. There have been cases of RT reported recently where they encounter similar difficulties in diagnosing the disease and the definitive diagnosis was made either by taking a core or surgical biopsy [[16], [17], [18]].

Likewise, the clinical features; there is no specific pattern of laboratory investigation results that are peculiar to the RT. Our patient demonstrated the manifestations of multiple endocrine deficiencies at presentation most likely due to bilateral symmetric involvement and destruction of the thyroid and parathyroid glands. He had symptomatic hypothyroidism and hypoparathyroidism and was on Thyroxin, Calcium, and vitamin D. Papi and Schwaegerle have described that about a third of patients with RT are hypothyroid at presentation [19,20].

Kumar in his review of cases of RT from the year 2002–2018 have demonstrated that in almost all of the cases FNA fails to distinguish among Riedel's thyroiditis, thyroid malignancy and other benign thyroid conditions, so open neck surgery was often required [16]. Our patient underwent FNA twice from thyroid and once from cervical lymph nodes and all resulted in none conclusive results. FNA is the part of the initial assessment of any thyroid mass and should be done as routine but how helpful it is in diagnosing the RT is remains controversial. The disease is easily misdiagnosed due to low incidence and limited experiences for most clinicians. Therefore, we hold an opinion that surgical biopsy is still the key tool for the definite diagnosis of RT in that the presentation of RT may mimic thyroid malignancy.

When it comes to the management of RT, several agents are available without any consensus of opinion [21]. This is understandably so since the rarity of the disease makes it almost impossible to accumulate an adequate number of patients and conduct a clinical trial to know the flawless treatment options. However, high dose corticosteroids particularly prednisolone is effective when given as monotherapy or in combination with Levothyroxine [19,22]. The dose and duration of therapy should be based upon the response and tolerability. The usual dose requirement is 15–60 mg of prednisolone daily [21].

Those who do not respond to steroids develop side effects or experience recurrence on withdrawal; they may get benefits from Tamoxifen alone or in a combination of steroids [23]. Tamoxifen effect is believed to act through modulation of TGF-B stimulation, a potent inhibitor of fibroblast proliferation. In females, the Tamoxifen toxicity like the development of hot flushes and endometrial hyperplasia has provoked its substitution with Raloxifene, which is preferred by the most physician based on the excellent result produced by its use [24]. Our patient was given a combination therapy of Prednisolone 40 mg and Tamoxifen 20 mg daily along with Levothyroxine and Vitamin D supplements with a satisfactory and sustained response. Until the one year of follow up no toxicity was noted.

Rituximab is a monoclonal antibody against protein CD20, primarily found on the surface of B lymphocytes. More recently it has been used and approved for the treatment of refractory IgG4-RSD to standard treatment [25]. It has also been successfully utilized for the treatment of a refractory case of RT due to its strong association with IgG4 related disorders and similar etiology. Soh SB et all from the Alfred Health, Australia for the first time successfully used the Rituximab for the treatment of RT in a 42 years old female [26]. Following the same thought, Hunt L et al. from Sheffield Teaching Hospitals, UK has recently published a case of 45 years old female that remain refractory to corticosteroids and Tamoxifen for six years followed by a substantial sustainable response to Rituximab [27].

Surgery plays a limited role in the management of Riedel's thyroiditis and has been found ineffective and fraught with an abundance of complications when employed as a primary form of treatment. Our patient had to undergo surgery being symptomatic due to pressure effects and because of diagnostic difficulties. In all most all of the cases that have been reported in the literature so far, the patient underwent some form of surgical intervention, either incisional biopsy, hemithyroidectomy, subtotal or total thyroidectomy. So our case is not odd to the conventional practice.

As we know that the natural history of Riedel's thyroiditis is often that of progression, although it may stabilize or even regress spontaneously. However, relapse which may even be extra-thyroid is a common following withdrawal of medications. A third of patients will develop other fibrosing disorder over 10yrs but fortunately, the disease-specific mortality is rare [19]. The patient in this report responded to Prednisolone and Tamoxifen with a sustained response. However, the patient has only been followed-up for two years with almost complete resolution of the neck swelling. Closer and longer follow-up is necessary, especially upon withdrawal of medications, to see if there will be relapse or symptoms of extrathyroidal fibrosis will develop.

## 4 Conclusion

In conclusion, clinicians should be aware of RT; despite its rarity, it should be suspected in patients presenting with a hard thyroid mass with compressive symptoms. Differentiation of RT from other thyroid disorders, especially malignant lesions, is vital. Medical management like high dose glucocorticoids followed by Tamoxifen should be used to control the inflammatory fibrotic process. Optimal dose and duration of anti-inflammatory treatment need to be studied and needs further research. Surgical intervention should be restricted to obtain tissue for diagnosis and to rule out malignancy. Thyroidectomy is indicated for patients with compressive symptoms, suspicious malignancy, and failure of medical management. Extensive surgery due to the potential risk of complications should be avoided.

## Ethical approval

Ethical approval was taken from Institutional review board (IRB) IRB # 18–079.

## Sources of funding

This research did not receive any funding from any resource.

## Author contribution

Dr. Alam ara Shafi: corresponding author, preparation of case report and collection of patient data, wrote discussion part of manuscript.

Dr. Nourah Bin Saad: preparation of photographs and helped in writing discussion.

Dr. Bandar AlHarthi: Edit the whole manuscript.

## Trial registry number

1. Name of the registry:

2. Unique Identifying number or registration ID:

3. Hyperlink to the registration (must be publicly accessible):

This is a case report which does not require a registry.

## Guarantor

Dr. Alam Ara Shafi.

## Consent

Written informed consent was obtained from the patient for publication of this case report and accompanying images. A copy of the written consent is available for review by the Editor-in-Chief of this journal on request”.

## Provenance and peer review

Not commissioned, externally peer reviewed.

## Declaration of competing interest

The author does not have any conflict of interest with any person or organization.
