# Supplementary material for: Riedel's thyroiditis as a diagnostic dilemma - A case report and review of the literature
Source: Ann Med Surg (Lond). 2020 Feb 25;52:5–9. doi: 10.1016/j.amsu.2020.02.006 (PMC7049561; doi:10.1016/j.amsu.2020.02.006)
Supplement: Multimedia component 1 [file mmc1.docx]

| **SCARE 2018 Checklist** | | | |
| --- | --- | --- | --- |
| **Topic** | **Item** | **Checklist item description** | **Page Number** |
| **Title** | **1** | **Riedel’s Thyroiditis as a diagnostic dilemma - A case report and review of the literature** | 1 |
| **Key Words** | **2** | Riedel’s thyroiditis, thyroid mass, anaplastic carcinoma, thyroid lymphoma, diagnostic challenges | 1 |
| **Abstract** | **3a** | Introduction —Riedel's thyroiditis is a rare inflammatory process which not only involves thyroid gland but also the surrounding vital structures. It may also be associated with various forms of systemic fibrotic disorders. The exact etiology is not known, but currently, the most favored view is that of a localized form of the systemic fibrotic process. We report a case of Riedel's thyroiditis in a male patient, highlighting diagnostic challenges and a rare presentation of hypocalcemia and mimicking thyroid lymphoma. Clinical knowledge of such a presentation of Riedel's thyroiditis would enhance our ability to make a speedy diagnosis | 1 |
|  | **3b** | Presenting complaint and investigations – A 35 years old man presented with diffuse goiter, symptomatic hypothyroidism, and hypocalcemia. There was a diffusely enlarged thyroid gland measured about 15cm and 10cm in transverse and vertical axis respectively with a smooth surface and normal overlying skin. There were multiple, small, firm and mobile enlarged cervical lymph nodes and carotid pulsation was palpable bilaterally |  |
|  | **3c** | After extensive laboratory and radiologic workup, he underwent thyroid isthamusectomy for pressure symptoms. The final pathology report confirmed the diagnosis of Riedel’s Thyroiditis for which he received medical treatment with satisfactory response. |  |
|  | **3d** | Conclusion — Riedel’s Thyroiditis should be suspected in patients presenting with a hard thyroid mass with compressive symptoms. Thyroidectomy is indicated only for patients with compressive symptoms, suspicious malignancy and failure of medical management. The mainstay of treatment is medical so extensive surgery due to the potential risk of complications should be avoided. |  |
| **Introduction** | **4** | Background – summarise what is unique or educational about the case. Give reference to the relevant surgical literature and current standard of care. The background should be referenced, and 1-2 paragraphs in length. | 1 |
| **Patient Information** | **5a** | Demographic details –35 years old Saudi man of Najran district of Saudi Arabiah. He is a business man. | **2** |
|  | **5b** | Presentation – Electively referred to the Endocrine Surgery Division of Surgical Oncology Department at King Fahad Medical City, Riyadh, as a case of diffuse goiter, symptomatic hypothyroidism, and hypocalcemia. |  |
|  | **5c** | The past history was not significant for any chronic illness or surgery. |  |
|  | **5d** | Other histories – He used to smoke one packet of cigarettes per day for the last 15 years. He was on Thyroxin 125 mcg daily, Alfacalcidol 1mcg daily and calcium supplements as per his primary care physician prescription. He was not known to have any allergies. His family history is not significant for any genetic disorder or malignancies. |  |
| **Clinical Findings** | **6** | Physical examination revealed a middle-aged well-nourished man with normal vital signs as per his age. There was a diffusely enlarged thyroid gland measured about 15cm and 10cm in transverse and vertical axis respectively with a smooth surface and normal overlying skin. There were multiple, small, firm and mobile enlarged cervical lymph nodes and carotid pulsation was palpable bilaterally. | 2 |
| **Timeline** | **7** | Inclusion of data which allows readers to establish the sequence and order of events in the patient's history and presentation (using a table or figure if this helps). Delay from presentation to intervention should be reported. | NA |
| **Diagnostic Assessment** | **8a** | Thyroid function test showed thyroid stimulating hormone (TSH) level of 0.903 IU/mL (reference: 0.4-4.0), a normal free thyroxin (fT4) level of 17 pmol/L (reference: 9-22.2). Full blood count result showed white blood cell count of 7,800 cells/cmm (reference: 4,000-11,000) with neutrophils 43% (reference: 40-70), lymphocytes 54% (40-60) and eosinophils of 3% (reference: <2), platelets count was 109 × 103/cmm (reference: 150,000-450,000) and packed cell volume of 41% (reference: 38-48). Antithyroid antibodies; Tg: 420 IU/ml (reference: 0-4.11) TPO: 567 IU/ml (reference: 0-5.61). IgG4 levels were normal.  Radiology [Figures 2, 3 and 4] demonstrated the nonspecific thyroid enlargement with cervical lymphadenopathy. Initial Fine-needle aspiration cytology (FNAC) from both thyroid lobes was inadequate. Repeat FNAC thyroid showed scanty lymphocytes likewise the FNAC from cervical lymph nodes. A clinical diagnosis of primary thyroid lymphoma was made but further immunohistochemistry staining and flow cytometry failed to confirm the clinical diagnosis of thyroid lymphoma. | 3 |
|  | **8b** | Diagnostic challenges – None conclusive laboratory results and FNAC results. |  |
|  | **8c** | Diagnostic reasoning – Differential diagnoses were thyroid malignancy, lymphoma and thyroiditis; based upon the clinical presentation of thyroid swelling with cervical lymphadenopathy. |  |
|  | **8d** | Prognostic characteristics when applicable (e.g. tumour staging or for certain genetic conditions). Include relevant radiological or histopathological images in this section. |  |
| **Therapeutic Intervention** | **9a** | Pre-intervention considerations – if there were patient-specific optimisation measures taken prior to surgery or other intervention these should be included e.g. treating hypothermia/hypovolaemia/hypotension in a burns patient, Intensive care unit treatment for sepsis, dealing with anticoagulation/other medications, etc. | 3 |
|  | **9b** | Interventions – describe the type(s) of intervention(s) deployed (pharmacologic, surgical, physiotherapy, psychological, preventive). Describe the reasoning behind this treatment offered. Describe any concurrent treatments (antibiotics, analgesia, anti-emetics, nil by mouth, Venous thrombo-embolism prophylaxis, etc). Medical devices should have manufacturer and model specifically mentioned. |  |
|  | **9c** | Intervention details – describe what was done and how. For surgery include details on; anaesthesia, patient position, use of tourniquet and other relevant equipment, prep used, sutures, devices, surgical stage (1 or 2 stage, etc). For pharmacological therapies include information on the formulation, dosage, strength, route, duration, etc. Include intra-operative photographs and/or video or relevant histopathology in this section. Degree of novelty for a surgical technique/device should be mentioned e.g. "first in human". NA |  |
|  | **9d** | Who performed the procedure – The procedure was performed by Consultant Endocrine surgeon with 2 years of specialized training and ten years of working experience in endocrine surgery. |  |
|  | **9e** | Changes – if there were any changes in the interventions, describe these details with the rationale. NA |  |
| **Follow-up and**  **Outcomes** | **10a** | Follow-up – He is being followed up by rheumatology, endocrinology, and ophthalmology. His current medications are, Thyroxin 125 microgram/day, Vitamin D 50000 IU/month, calcium carbonate 1 gm/day, Prednisolone 5 mg/day and Rivaroxaban 20 mg/day. | 3 |
|  | **10b** | Outcomes - Clinician assessed and (when appropriate) patient-reported outcomes (e.g. questionnaire details). Relevant photographs/radiological images should be provided e.g. 12 month follow-up. |  |
|  | **10c** | Intervention adherence/compliance – He had a good compliance to his medications. |  |
|  | **10d** | Complications and adverse events – all complications and adverse or unanticipated events should be described in detail and ideally categorised in accordance with the Clavien-Dindo Classification. How they were prevented, diagnosed and managed. Blood loss, operative time, wound complications, re-exploration/revision surgery, 30-day post-op and long-term morbidity/mortality may need to be specified. If there were no complications or adverse outcomes this should also be included. |  |
| **Discussion** | **11a** | Strengths – describes the strengths of this case | 4 |
|  | **11b** | Weaknesses and limitations in your approach to this case. For new techniques or implants - contraindications and alternatives, potential risks and possible complications if applied to a larger population. If relevant, has the case been reported to the relevant national agency or pharmaceutical company (e.g. an adverse reaction to a device) |  |
|  | **11c** | Discussion of the relevant literature, implications for clinical practice guidelines and any relevant hypothesis generation. |  |
|  | **11d** | The rationale for your conclusions. |  |
|  | **11e** | The primary “take-away” lessons from this case report. |  |
| **Patient Perspective** | **12** | Patient had a satisfactory response towards the treatment he received at the hospital. |  |
| **Informed Consent** | **13** | Written informed consent was obtained from the patient for publication of this case report and accompanying images. A copy of the written consent is available for review by the Editor-in-Chief of this journal on request. | 6 |
| **Additional Information** | **14** | Approval of the case report was obtained from the Institutional Review Board.  The author does not have any conflict of interest with any person or organization.  This research did not receive any funding from any resource. | 7 |
